# Recent Advances on Cellulose Nanocrystals and Their Derivatives

**DOI:** 10.3390/polym13193247

**Published:** 2021-09-24

**Authors:** Shuting Peng, Qiguan Luo, Guofu Zhou, Xuezhu Xu

**Affiliations:** 1 Guangdong Provincial Key Laboratory of Optical Information Materials and Technology and Institute of Electronic Paper Displays, South China Academy of Advanced Optoelectronics, South China Normal University, Guangzhou 510006, China; stpeng@m.scnu.edu.cn (S.P.); luoqiguan@163.com (Q.L.); 2 National Center for International Research on Green Optoelectronics, South China Normal University, Guangzhou 510006, China; 3 Shenzhen Guohua Optoelectronics Tech. Co. Ltd., Shenzhen 518110, China; 4 Academy of Shenzhen Guohua Optoelectronics, Shenzhen 518110, China

**Keywords:** nanocellulose derivatives, cellulose nanocrystals, surface modification, polymerization, functional materials, small molecules, macromolecules, polymers

## Abstract

Nanocellulose, typically cellulose nanocrystals (CNCs), has excellent properties and is widely used. In particular, CNC has a small dimension, high chemical reactivity, and high sustainability, which makes it an excellent candidate as a starting material to be converted into nanocellulose derivatives. Chemical modification is essential for obtaining the desired products; the modifications create different functional attachment levels and generate novel microstructures. Recent advances on nanocellulose derivatives have not yet been reviewed and evaluated for the last five years. Nanocellulose derivative materials are being used in a wide variety of high-quality functional applications. To meet these requirements, it is essential for researchers to fully understand CNCs and derivative materials, precisely their characteristics, synthesis methods, and chemical modification approaches. This paper discusses CNC and its derivatives concerning the structural characteristics, performance, and synthesis methods, comparing the pros and cons of these chemical modification approaches reported in recent years. This review also discusses the critical physicochemical properties of CNC derivative products, including solubility, wetting performance, and associated impacts on properties. Lastly, this paper also comments on the bottlenecks of nanocellulose derivatives in various applications and briefly discusses their future research direction.

## 1. Introduction

**Figure 1:**
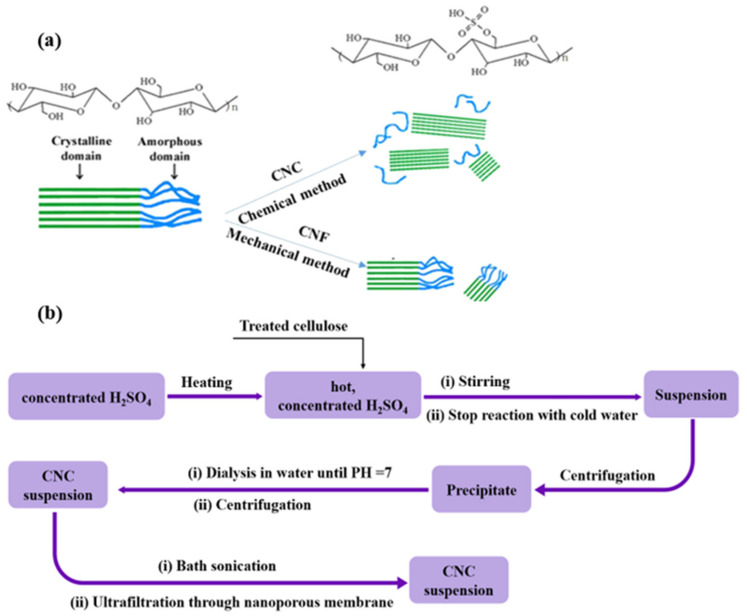
(**a**) Schematic representation of sulfuric hydrolysis of cellulose nanocrystals. (**b**) Diagram of the process of extracting CNC from cellulose-containing raw materials.

Cellulose is the most abundant polymer material in nature. It is widely present in plant cell walls, microbial secretions (*Gluconacetobacter xylinus*) [1], and tunicates [2]. Cellulose is a natural linear polymer (polysaccharide). It is composited of repeating anhydro-D-glucose units, joined by β-1 → 4 glycosidic oxygen linkages. Each repeating unit is rotated 180 degrees around the axis of the cellulose backbone in comparison with the adjacent ring. The regular arrangement of the hydroxy groups along the cellulose chain leads to the formation of H-bridges, which in turn leads to a fibrillar structure with crystalline properties. Specifically, cellulosic chains are rearranged into different regions: the ordered crystalline and the disordered amorphous ones [3]. Indeed, the cellulose chain consists of two parts: amorphous regions and crystalline regions (See Figure 1a). The molecular arrangement in the amorphous region is loose, and nanocellulose derivatives can be obtained by breaking the amorphous region of the cellulose chain, for example, the preparation of CNC by acid hydrolysis.

CNC presents in the form of nanorod, nanowhiskers, and rod-like particles. They are characterized as sustainable and eco-friendly nanomaterials. CNCs possess unique properties of high aspect ratio, high surface area, high mechanical strength, and liquid crystalline nature. CNC possesses a relatively low aspect ratio; it has a typical diameter of 2–20 nm and wide length distribution from 100 to 600 nm [4,5]. The size, morphology, and crystallinity of CNC are affected by the cellulose source and preparation method [3,4,5,6,7]. CNC can have a crystallinity level above 70% (Table 1) The high degree of crystallinity is responsible for the higher rigidity. Most importantly, CNC materials can contain sulfate, hydroxyl, or carboxyl groups on their surface, making them applicable for further functionalization by polymers, catalysts, or dye molecules, imparting to CNCs new characteristics and properties expanding the scope of their application [8].

The excessive proliferation of non-degradable polymers is a severe environmental problem. Thus, paying attention to cellulose and its derivatives is indispensable since these natural polymers are rich in sources and degradable. Due to the small size, large specific surface area, and high content of surface hydroxyl groups, the drying of aqueous dispersions of CNCs causes the aggregation of nanocrystallites, which leads to the formation of microcrystalline materials instead of nanomaterials [9]. Fortunately, this problem can be solved well by chemical modification. It must be mentioned that many modifications of CNCs proved to be effective to suit theirs as a functional part of applications. However, key issues such as the stability of surface chemical modification, thermoforming process, and compatibility of modified CNCs with the composite matrix need to be deeply studied. Since the superior performance of nanomaterials can be achieved through the chemical modification of nanocellulose fillers, the methods and types of surface modification of CNCs are attracting great attention [10,11].

According to the Web of Science database report, ~38 reviews were associated with nanocellulose derivatives in 2017–2021. As we refined the search for surface modification, we found 10 reviews on this specific topic. After we excluded the specific application in carbon capture, drug carriers, wound healing, antibacterial materials, and supercapacitors, we found that Neves et al. have reviewed the recent advances in 2021 [12] on modified cellulose/nanocellulose epoxy composites. Lucia et al. have reported, in 2020 [13], the structural reconstruction strategies for cellulose nanomaterials and functional properties. In 2018, Berglund et al. have also reviewed the processing for structured cellulose nanocomposites [14]. The reviews mentioned above focused on the preparation of nanocomposites and properties, rarely discussing nanocellulose derivatives in the sense of their chemical surface modification methods.

In this review, the surface chemical modification methods of CNCs are generally summarized into two categories: (1) Chemical modification using small molecules, e.g., esterification, silylation, cationization, treatment with isocyanates, and so on. (2) Grafting macromolecules on the CNC surface. We will focus on the methodologies of various surface modifications on CNCs. Different molecular sizes have different roles, such as feasibility, particle-particle interaction, and target application. This paper introduces the current modification strategies and the changes in physicochemical properties and performance of cellulose nanocrystals. Here, it also involves a wide range of derivative materials from polymer reinforcements to biomedical materials and optoelectronic functional materials. 

## 2. Isolation of CNCs

CNCs are facile and to be made from plants and agricultural residues such as bamboo [15,16], eucalyptus [17], root jute fiber [18], root coir fiber [19], pineapple leaf [20], straw [21], potato peel [22], and so on. CNCs are obtained by applying a chemical treatment to cellulose fibers [23]. Inorganic acid hydrolysis is the most commonly used method to prepare CNC. In recent years, to pursue efficient and environmentally friendly preparation methods, various new preparation methods have been developed. For example, organic acid hydrolysis [24], solid acid hydrolysis [25], ionic liquid [26], TEMPO oxidation [27], acid vapor [28], eutectic solvent [29], and Pickering emulsion oxidation [30]. Since different preparation methods can affect the physical and chemical properties of CNC, different preparation methods of CNC can be selected according to the properties of different raw materials, or appropriate preparation methods can be selected according to the different uses of CNC.

### 2.1. Inorganic Acid Hydrolysis

**Figure 2:**
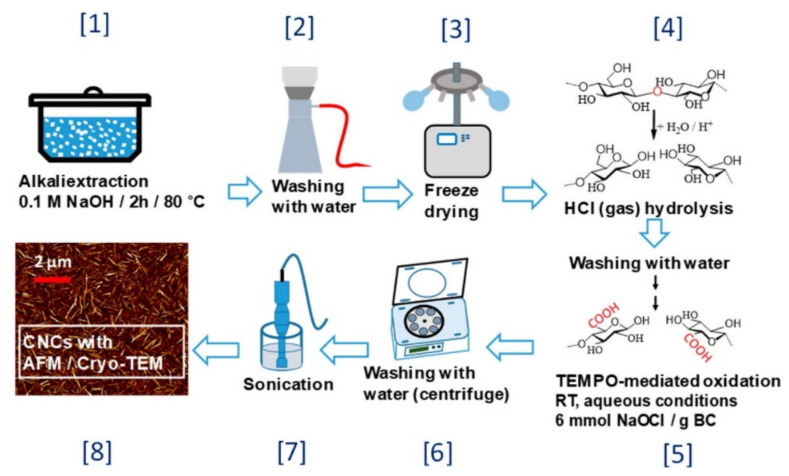
Scheme of the CNC production process [34]. Copyright © 2019 American Chemical Society.

Inorganic acids (such as sulfuric acid [31], hydrochloric acid [32], hydrochloric acid/nitric acid mixed acid [33], and phosphotungstic acid [25]) can be used to prepare CNCs. Due to the fact that cellulose has both crystalline and amorphous regions, the orientation and arrangement of amorphous domains are random, resulting in a loose structure in these domains, which makes acid easily “dissolve” the domains and release nanocrystals [23]. The suspension of CNCs prepared by sulfuric acid hydrolysis has high stability but poor thermal stability. Compared with sulfuric acid, phosphotungstic acid is easier to process and recycle, but the reaction time is longer [25]. The acid steam method can also be considered as inorganic acid hydrolysis. It is through heating HCl molecule to adsorb it on the surface of cellulose, and then hydrolysis reaction occurs after combining with water on the cellulose surface, which can effectively remove the amorphous region. It can also be combined with other methods. For example, Kontturi et al. pretreated bacterial cellulose with NaOH for two hours at 80 °C and prepared CNC with a length of 100–300 nm and a yield of about 80% after freeze-drying by HCl steam hydrolysis combined with TEMPO oxidation and ultrasonic treatment (Figure 2) [34].

Inorganic sulfuric acid hydrolysis is proved to be the earliest and most frequently used method to prepare CNC suspensions [35] (Figure 1). Different researchers proposed various hydrolysis conditions: concentration of H_2_SO_4_ can vary from 40 to 65%, temperature from 40 to 80 °C, and time from several minutes to several hours. The entire reaction process is often accompanied by stirring. Indeed, optimization and control of acid hydrolysis have been the subject of several publications. If common parameters are the hydrolysis with 64 wt% sulfuric acids at 40–45 °C for about 30 min, it has been proved that variation of one of the parameters can largely influence the reaction yield and CNC properties [36]. After hydrolysis, the hydrolyzed cellulose-containing aggregates of CNCs are obtained. In addition, a step of ultra-sonication should be applied for isolate free CNCs. Finally, dialysis and further filtration through a nanofilter to remove big particles should be carried out to obtain homogenous CNCs (Figure 1). Lastly, slow evaporation by water bath heating or rotating evaporation of obtained dilute CNCs suspension is used to increase the concentration of CNCs [37,38]. In addition, CNC particles can be obtained by freeze-drying, spray drying, or spray freeze-drying. Khoshkava et al. have studied the effect of the above drying techniques, including spray drying, freeze-drying, and spray freeze drying, on the microstructure of CNC particles [39]. The study found that CNC microstructure depends highly on employed drying techniques. Freeze drying produces larger particles with a flake structure, while smaller particles with spherical shapes are produced by spray drying. Spray freeze drying of CNC suspension resulted in a powder with a porous structure.

**Figure 3:**
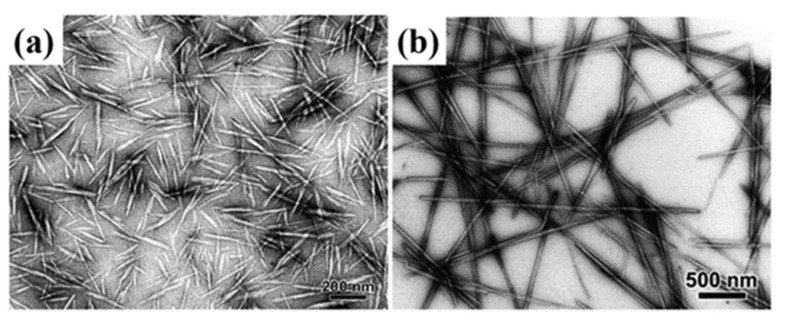
TEM photos of cellulose nanocrystals from different sources. (**a**) Hydrolysis with sulfuric acid ramie [44]; (**b**) hydrolysis with sulfuric acid of ascidian cellulose [45].Copyright © 2008, American Chemical Society.

The length of CNCs prepared by acid hydrolysis of cellulose is generally tens to hundreds of nanometers, and the cross-sectional diameter is approximately three to tens of nanometers (Figure 3). This process imparts abundant anionic sulfate ester groups (–O-SO_3_^−^) onto the CNCs surface, introducing electrostatic repulsion of CNCs particles in aqueous dispersion, yielding stable CNCs over a wide pH range [40]. The hydrolysis conditions, including acid/fiber ratio, temperature, hydrolysis time, acid concentration [41], and ultrasonic time, form CNCs with different attributes of yield, sulfur content (including sulfuric acid), ion sites, Zeta potential, width, length, and aspect ratio (Table 1) [42,43]. 

**Figure 4:**
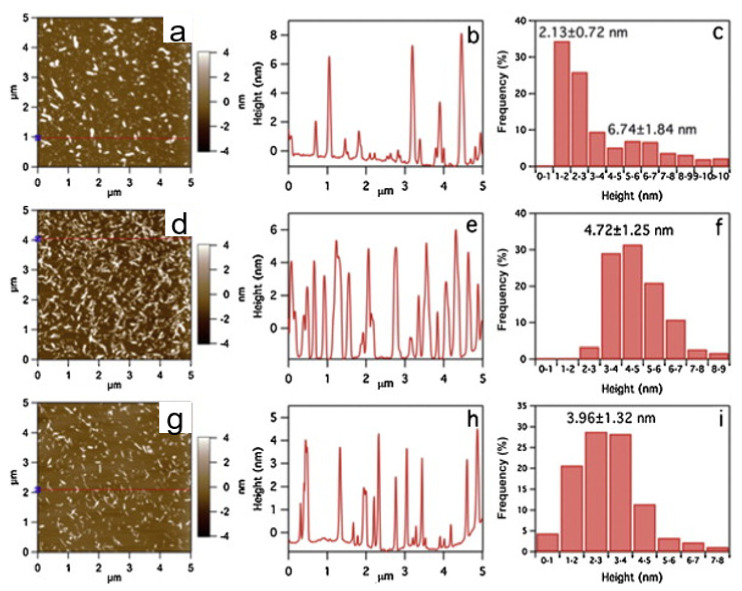
AFM of CNC hydrolyzed for 15 min (**a**–**c**), CNC hydrolyzed for 45 min (**d**–**f**), and CNC hydrolyzed for 60 min (**g**–**i**): (**a**,**d**,**g**) height images, (**b**,**e**,**h**) height profiles along lines in (**a**,**d**,**g**), and (**c**,**f**,**i**) lateral dimension distribution [38].Copyright © 2013 Elsevier Ltd. All rights reserved.

Taking the acid hydrolysis of rice straw to obtain CNCs as an example, Hsieh et al. [29] set time as a single variable and studied its effect on the dimension of CNCs. They found that 15 min of hydrolysis time generated both nanorods and abundant nanoparticles; see a (15 min), 4d (45 min) and 4g (60 min). AFM height profiles and distribution showed CNC15 nanoparticles and nanorods as 2.13 ± 0.72 nm and 6.74 ± 1.84 nm thick, respectively (Figure 4b,c). Longer hydrolysis reduced the average thickness of CNCs, respectively. As the hydrolysis progressed, the nanorods reduced in lateral dimensions while the smaller nanoparticles were broken down to soluble mono- and/or oligo-saccharides. Lengthening the hydrolysis time to 60 min reduced both nanorod size and yield further. The above studies showed that conditions of the hydrolysis process firmly determined the characteristics, especially the length and width. However, due to low productivity and high expenses, it is still difficult to start the industrial manufacturing of CNCs, which is one of the bottlenecks limiting the widespread obtaining and use of CNCs.

### 2.2. Other Methods

CNC prepared by organic acid hydrolysis has the advantages of high yield, high thermal stability, and recycling of organic acids, but the processing time is extended. Organic acid treatment is usually combined with other methods. For example, Xu et al. pretreated bamboo slices with 85% formic acid for 45 min and then treated them with the TEMPO oxidation system for 0.5–24 h to obtain CNC with the length of 80–300 nm and width of 5–9 nm [51].

The eutectic solvent method was first reported by Abbott et al. in 2001. Deep eutectic solvent is a novel hydrolysis strategy of nanocellulose, composed of hydrogen bond acceptor and hydrogen bond donor to form two or three components of hypoeutectic mixture. Microwave-assisted ultrasonic processing can effectively promote the deep eutectic solvent pretreatment process, realizing the efficient preparation of CNC. In addition, deep eutectic solvent can also be effectively recycled [29].

Recently, more and more new methods for preparing CNC have been continuously reported. Zhang et al. used microcrystalline nanocellulosic materials to form Pickering emulsion in a mixture of n-hexylamine, n-hexane, and sodium iodate aqueous solution. Then, CNCs were finally obtained with the length of 120.1 ± 7.9 nm and the width of 4–8 nm after two weeks of light-protected reaction [30]. American Process Company developed the AVAP technology, using SO_2_ and ethanol to remove hemicellulose, lignin, and amorphous cellulose regions from raw materials [52]. The key aspect of the AVAP technology is that the pretreatment process can be controlled, and different forms of nanocellulose can be obtained by controlling reaction conditions and combining them with subsequent processing methods.

## 3. Surface Modification of CNCs

The surface modification of CNCs can not only retain the original properties of CNCs, but also give new surface properties, such as hydrophobic [53,54], biocompatibility [55], antistatic properties [56,57], and dyeing properties [58]. The surface of the CNCs contains numerous hydroxyl (–OH) groups, which provide the main reaction site for modification. Esterification, etherification, oxidation, silylation, and grafting of macromolecules are typical ways. Table 2 lists some representative methods of functional modification on both the surface and the terminal end of CNC that currently exist. [59] We listed the findings, advantages, and limitations of the approaches. All these surface modification methods are based on the following three aims: (1) To reduce the size of CNCs in dispersions with organic solvents by increasing the hydrophobicity of the surface of nanocrystallites; (2) to improve compatibility between CNCs and hydrophobic polymers matrix; and (3) to endow CNC additional attributes, such as biology, optics, mechanics, and electromagnetics, by covalently bonding with functional macromolecules [60]. In this article, we will detail the approaches mentioned earlier.

### 3.1. Small Molecules Chemically “Grafted to” CNCs

The simplest way to change the surface properties of CNCs is to attach small molecules of various reagents. Mostly, hydroxyl groups on the surface of CNCs are converted into hydrophobic groups of small molecules through modification, which will change the CNCs’ dispersion state, charge amount, and type, as well as other features of nanoparticles. 

#### 3.1.1. Esterification

**Figure 5:**
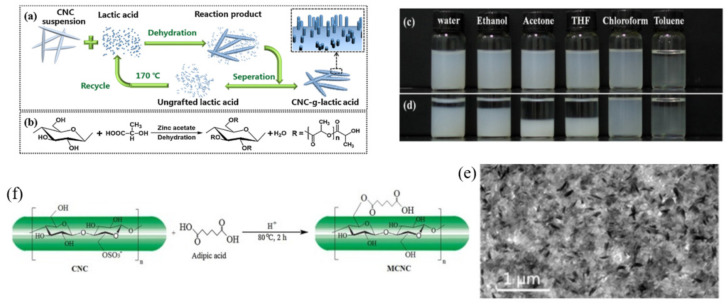
(**a**) Schematic illustration of the preparation of CNC grafted with lactic acid (CNC-g-LA) and the recycling of ungrafted lactic acid; (**b**) reaction equation of the esterification reaction on the surface of CNC. Photographs of CNC-g-LA suspensions in different solvents after one min sonication (**c**), and after 24 h standing (**d**); (**e**) cross-sectional TEM image of nanocomposite containing 3 wt% of CNC-g-LA. (**f**) Surface modification with adipic acid of CNC [61,63]. © 2018 Elsevier Ltd. All rights reserved. for [61], © 2017 Elsevier B.V. All rights reserved. for [63].

Lin et al. used polylactic acid (PLA) and microcrystalline cellulose (MC) fibers as raw materials to prepare a high-performance bio-based green composite material L-lactic acid oligomer (g-MC) [61]. Inspired by this, an example of an esterification reaction is a modification of CNCs with lactic acid (CH_3_CHOHCOOH) proposed by Wu et al. [62], who claimed that it is a green and simple one-step process. Their modified CNCs were wrapped by a small number of lactic acid (Figure 5). The attachment strategy of small hydrophobic molecules can improve the surface graft density and the graft length of modified CNCs.

It has been reported that over 87% of all available –OH groups on the surface of CNCs were replaced with lactic acid, successfully overcoming the low grafting rate. The modified CNCs grafted with lactic acid (CNC-g-LA) formed stable dispersions in chloroform. However, dispersions of CNC-g-LA particles in water, ethanol, acetone, THF, and toluene were unstable and were separated after 24 h (Figure 5d). Similarly, Ferreira et al. reported that CNCs from bagasse pretreated with organic solvents could be modified with adipic acid at low temperatures [63]. The reaction is also completed in one step. The resulting product has increased hydrophobicity and can also be well dispersed in chloroform. This strategy improves the hydrophobicity and dispersion state of CNCs in various organic solvents, proving that small molecular modification is helpful for achieving a good hydrophobicity. It is beneficial to adjust the interface interaction between CNCs and matrix polymers if we use CNCs as a reinforcer.

**Figure 6:**
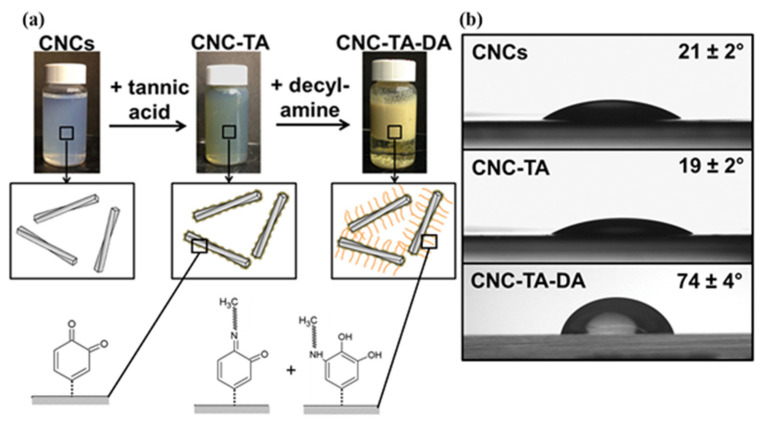
(**a**) Surface modification of CNCs with tannic acid followed by decylamine addition; (**b**) water contact angles (measured after 30 s) for CNCs, CNC-TA, and CNC-TA-DA films produced by spin coating [64].

Recently, low-cost plant polyphenols such as tannic acid have been used as precursors to form multifunctional coatings. Polyphenols can be used as surface small molecule modification of CNCs. Hu et al. [64] proposed a hydrophobic modification method for CNC nanomaterials. First, tannic acid-coated CNCs (CNC-TA) were prepared via oxidation and oligomerization of tannic acid on the surface of CNCs nanoparticles. Next, decylamine and tannic acid were coated on CNC-TA particles by Chiff base formation/Michael-type reaction, thus achieving hydrophobic modification (Figure 6a). Chiff base formation/Michael-type addition covalently attaches primary amines with long alkyl tails to CNC-TA, which increases the particles’ hydrophobicity (contact angle shift from 21 to 74°) (Figure 6b). A low increment of contact angle is due to the presence of hydroxyl groups on the foreign molecules.

**Figure 7:**
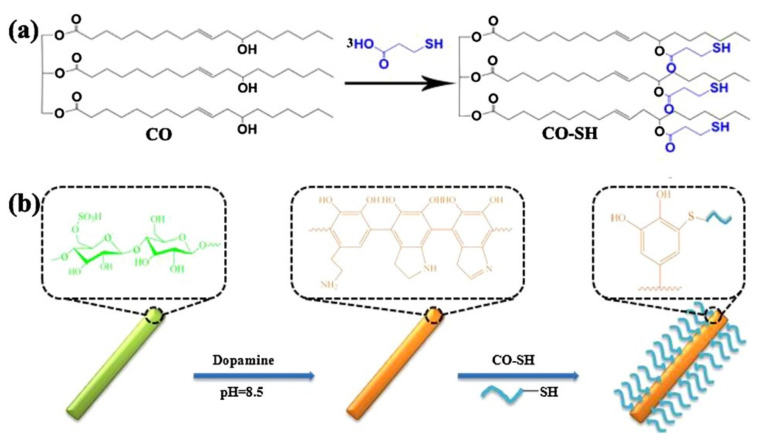
(**a**) Synthetic route of CO-SH; (**b**) schematic illustration of the strategy for synthesizing CNC/polydopamine/CO [65]. © 2018 Elsevier Ltd. All rights reserved.

While the hydrophobic property and dispersity of modified CNCs have significantly been improved as described in recent literature, the utilization of synthetic products obtained from a petrochemical feedstock for the surface modification of CNCs still brings about environmental issues. Plant oils with long hydrophobic hydrocarbon chains of triglycerides have been developed as alternatives to non-renewable resources. Inspired by mussel adhesive protein, Shang et al. proposed an eco-friendly approach for hydrophobic modification of CNCs (Figure 7) [65]. For this, the CNCs were functionalized with polydopamine via a spontaneous polymerization of dopamine. Secondly, the hydrophobic modification process was performed through the Michael addition reaction between the catechol group in the polydopamine and the thiol-containing castor oil (CO-SH). The latter was presynthesized by the esterification reaction between castor oil and mercaptopropionic acid. The modified CNCs preserved cellulose crystallinity, displayed higher thermal stability than unmodified CNCs, and were highly hydrophobic with a water contact angle of 95.6°.

**Figure 8:**
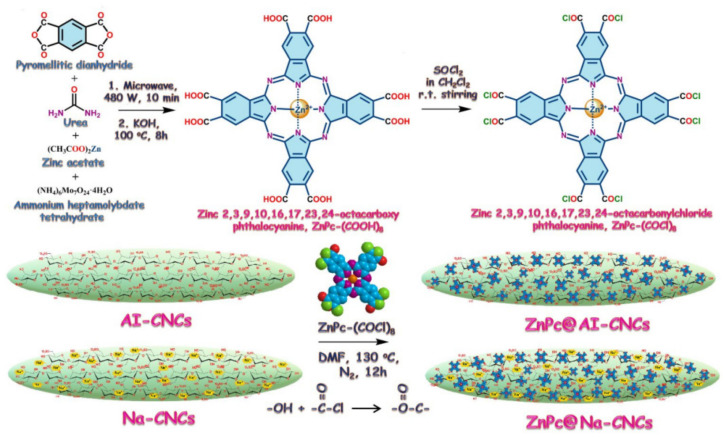
Step 1: Synthetic protocol of zinc octacarboxyphthalocyanine, ZnPc(COOH)8. Step 2: Transformation of ZnPc(COOH)_8_ to activated zinc octacarbonylchloride phthalocyanine. Step 3: Covalent functionalization of AI-CNC, Na-CNC with ZnPc via ester linkage to form ZnPc-CNC conjugates [66]. Copyright © 2020, American Chemical Society.

A zinc phthalocyanine conjugate was attempted by Kazi Alam et al. Covalent esterification of CNCs with ZnPc was achieved by taking advantage of the abundant -OH functional groups on CNCs and activated -COCl group on ZnPc-(COCl)_8_ (Figure 8), which react together to form ester linkage (-COO-) at 130 °C for 12 h under 4 h nitrogen atmosphere [66]. The product of zinc phthalocyanine conjugated to CNCs displays fluorescence. The conjugated CNCs show bright green and exhibited absorption and emission maxima at 690 and 715 nm. Moreover, the authors inferred that zinc phthalocyanine is mostly located on the surface of Na-CNC in a face-down manner and rigidly binds to Na-CNC through ester bonds. In ZnPc @ AICNC, more metal phthalocyanine rings may be oriented perpendicular to the surface (boundary connection to the CNCs surface). This protocol makes CNCs to be readily used to implement photoluminescence-based technologies, such as fluorescence microscopy, total internal reflection fluorescence imaging, fluorescence lifetime imaging microscope, and so on.

#### 3.1.2. Isocyanate

**Figure 9:**
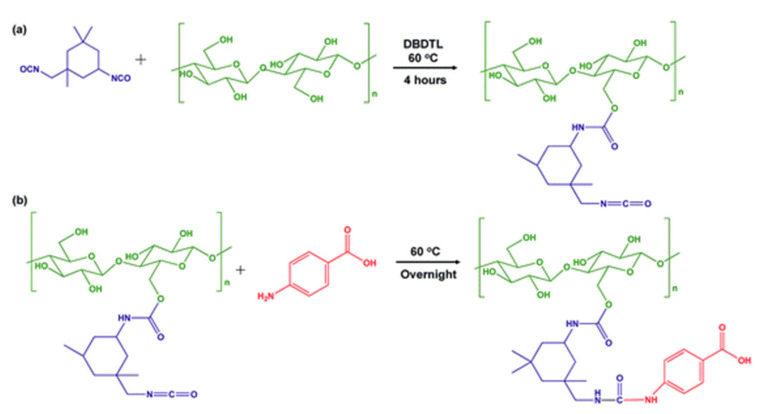
Reaction scheme of (**a**) two-step modification of CNCs’ surface using IPDI to produce CNC–IPDI intermediate product and (**b**) attachment of PABA to modified CNCs to produce CNC–IPDI–PABA [67]. Copyright © 2018, American Chemical Society.

**Figure 10:**
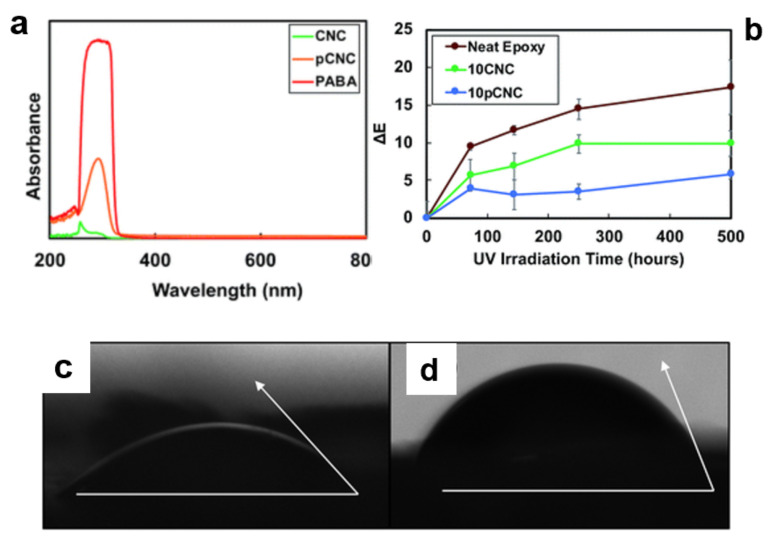
UV absorbance of (**a**) CNCs, PABA, and CNC–IPDI–PABA. (**b**) Discoloration of selected samples after exposure to UV radiation for a prolonged period. Contact angles of water on (**c**) native CNCs, and (**d**) CNC–IPDI–PABA [67]. Copyright © 2018, American Chemical Society.

A coupling reaction can be beneficial to attaching unreactive molecules to the surface of CNCs. For example, Panchal et al. used the isophorone diisocyanate (IPDI) to graft a UV-filter molecule such as p-aminobenzoic acid (PABA) to the surface of CNCs. In this reaction, IPDI was used as a coupling agent (Figure 9) [67]. The coupling efficiency of the whole process is 8%. However, the final product exhibits excellent UV absorption characteristics. The hydrophobicity of CNC-IPDI-PABA is 98% higher than that of CNCs (Figure 10). Despite the above benefits, there are relatively few coupling agents, which can connect functional molecules to CNC because of the lack of convenient coupling agents and a suitable solvation system.

**Figure 11:**
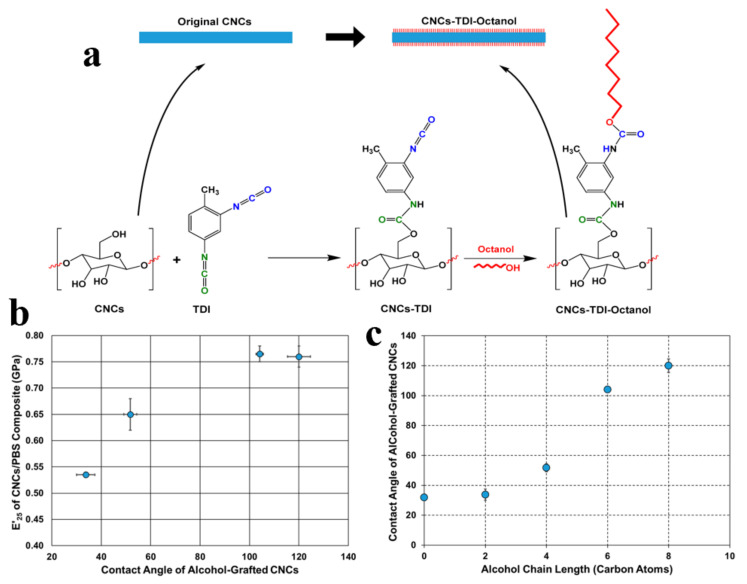
(**a**) Approach to tailor the hydrophilicity of CNCs by grafting alcohols of different chain lengths on the CNCs’ surface using TDI as a linker. (**b**) The relationship between the contact angle of the alcohol-grafted CNCs and their capabilities to reinforce PBS. (**c**) The dependence of the contact angle of the modified CNCs on the chain length of the alcohols grafted onto their surfaces [68]. Open access.

Another example of reacting isocyanate and CNCs was reported by H. Abushammala et al. who highlighted a successful attachment of different alcohols (ethanol, 1-butanol, 1-hexanol, and 1-octanol) on CNCs [68]. Grafting the alcohols with different chain lengths changes CNC’s hydrophilicity dramatically. The authors chose 2,4-toluene diisocyanate (DTI, C_9_H_6_N_2_O_2_) with the surface hydroxyl groups’ CNCs and hydroxyl groups of alcohols, respectively. The contact angle of water on the modified CNCs increased significantly from 32° to 120°, which implied the evolution of a hydrophobic alkyl-based shell around the CNCs (Figure 11c).

#### 3.1.3. Triazinyl

**Figure 12:**
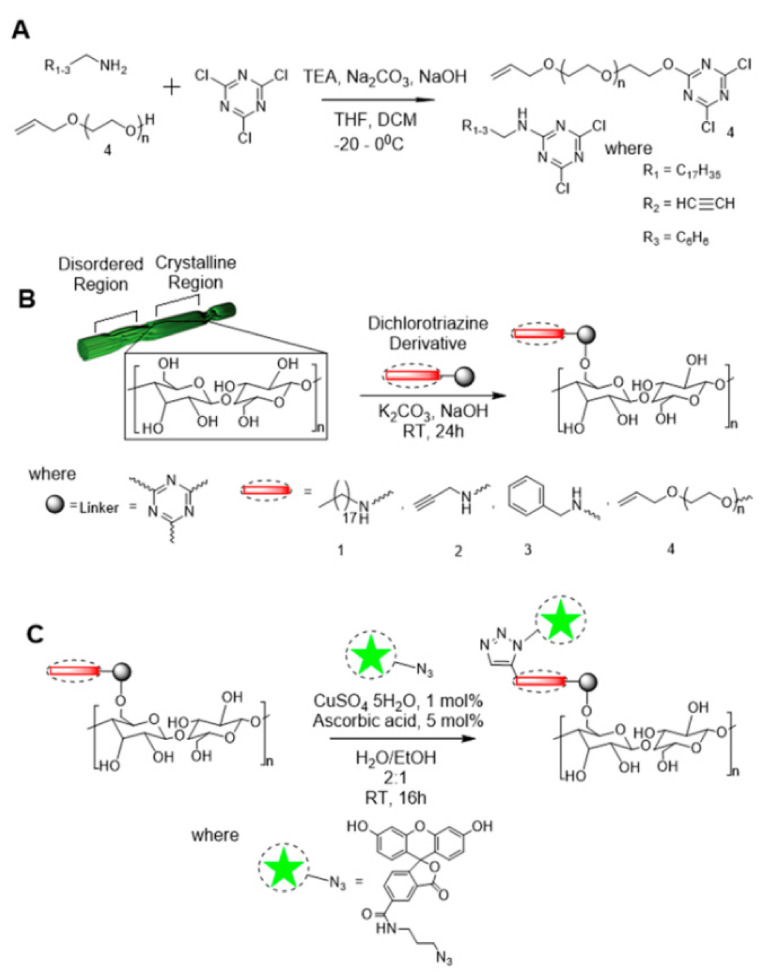
(**A**) Synthesis of 4,6-dichloro-1,3,5-triazine derivatives, (**B**) chemical grafting of 4,6-dichloro-1,3,5-triazine derivatives onto cellulose, and (**C**) fluorescein azide click grafting onto alkyne-modified nanocellulose [69]. Copyright © 2018 American Chemical Society.

Fatona et al. reported that target molecule triazine derivative (small molecule derivatives) was grafted onto the CNCs to obtain fluorescent ones with good stability in water, methanol, ethanol, and propanol (Figure 12) [69]. In this strategy, triazine chemistry was used, and the triazine-based linker reacted with hydroxyl-rich CNCs (Figure 12A). 

**Figure 13:**
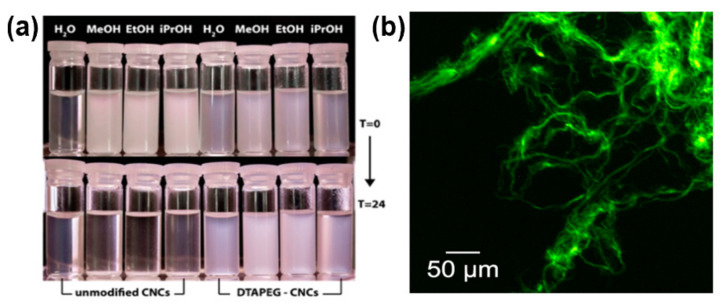
(**a**) Photographs of unmodified and modified CNC suspensions (0.5 wt%) in aqueous and organic media at T = 0 h and T = 24 h after sonication; (**b**) DTP-grafted BMCC fibrils labeled through a secondary azide-alkyne cycloaddition reaction with fluorescein derivatives [69]. Copyright © 2018, American Chemical Society.

The advantage of this strategy is that the selective chlorine substitution of cyanuric chloride can be controlled with moderate temperatures, making it a highly predictable procedure for nanocellulose modification. The fluorescent cluster was also quickly clicked to the terminal of triazine through a “click reaction.” Surprisingly, the modified CNCs displayed high colloidal stability in the comparatively extensive range of both aqueous and organic solvents (Figure 13).

Frequently, a surface modification of CNCs will cause a degree of aggradation when replacing the solvent in CNC suspension, which leads to detectable micron-sized bundles visible in TEM microscopy. The CNCs modified with highly hydrophobic groups such as fluorinated end group can be dispersed in dimethylformamide and fluorine-containing solvents, but the dispersion state is not optimistic. This type of reaction gives micron-sized aggregates that are made of several individual CNCs. In this representative triazine chemistry article, we would see the chance of obtaining other functional CNCs’ derivatives without harming its original attributes, as it happens in its original aqueous environment. Moreover, as the authors pointed out, this procedure can act as the basis for secondary modification, designing hetero-bifunctional triazinyl derivatives where one function can be used for solvent compatibilization and the second for targeted reactions.

#### 3.1.4. Cationization

**Figure 14:**
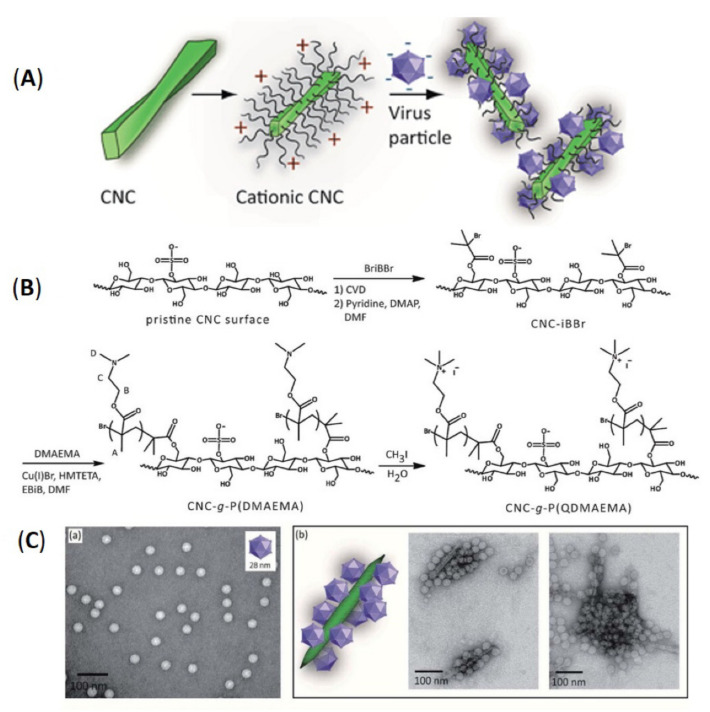
(**A**) Diagram of the proposed mechanism. (**B**) Reaction scheme for CNC surface modification, including the two-step initiator modification, SI-ATRP of DMAEMA, and quaternization of poly (DMAEMA) grafts on the CNCs. The letters A–D by the poly (DMAEMA) refer to the signals of the ^1^HNMR spectrum. (**C**) (a,b) TEM micrographs of virus and their complexes with CNC attached with poly (QDMAEMA) [70]. Royal Society of Chemistry; RSC Pub.

Cationization of CNCs such as attachment of N^+^-rich groups is a meaningful modification approach for the surface of CNCs to make it capable of catching anion-containing molecules. Of course, CNCs are anionic particles since they carry a large amount of sulfate ester group (–O-SO_3_^−^); therefore, the cationization of CNCs is a more complicated process. Rosilo et al. grafted the poly (2-(dimethylamino)ethyl methacrylate, DMAEMA, C_8_H_15_NO_2_) on the CNCs surface, which was then activated with methyl iodide to generate permanent cationic CNC-attached with poly (QDMAEMA) [70]. The poly (DMAEMA) itself is a stimuli-responsive polycation (Figure 14). The cationic CNCs have a solid affinity to anionic virus particles at different salt concentrations. Therefore, the virus particles can be concentrated and extracted from the solution by centrifugation. Though this process has disadvantages, it made CNCs go through strict organic solvent infiltration steps and the cleansing environment by the atom transfer radical polymerization (ATRP), though this strategy appears as a novel way to control the charges on the CNCs.

**Figure 15:**
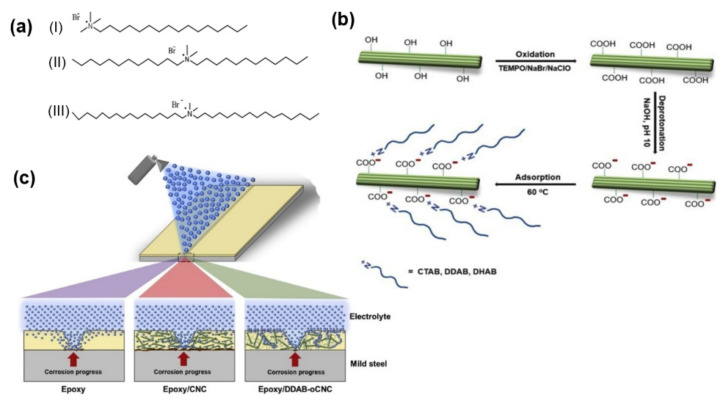
(**a**) The molecular structures of the cationic surfactants. (**b**) Schematic representation of surfactant modification of CNCs using a three-step process. (**c**) Schematic representative of hydrophobic modified CNCs nanocomposite coatings [71]. © 2019 The Korean Society of Industrial and Engineering Chemistry. Published by Elsevier B.V. All rights reserved.

The above case is carried out via graft polymerization of a cationic polymer on CNCs. Except that, there is another physical mixing method to introduce surfactants to CNC directly. Malinly et al. attempted to use several types of cationic (-N^+^) surfactants (e.g., cetyl tetramethyl ammonium bromide (CTAB, C_16_H_33_(CH_3_)_3_NBr), to make it adsorb on the CNCs’ carboxyl groups (Figure 15) [71]. This strategy increased the hydrophobicity of CNCs and increased the dispersion ability of CNCs in the epoxy polymer matrix. The non-covalent physical adsorption of surfactants on nanocellulose is easier to control and manipulate than fine chemical modification [72]. It was proved that CNCs modified with hydrophobic molecules are suitable for manufacturing renewable and green coating compositions.

From the perspective of this review, the tuning of the interfacial properties of CNC is practical, as proven by Rosilo, Malinly, and Chen et al. respectively [70,71,73]. Still, one disadvantage is that the physical absorption of surfactants on CNC may encounter a problem, i.e., while the surfactant/CNC solids are loaded in an aqueous environment, the surfactant may leak easily. Nevertheless, the surfactant modification on CNCs is an essential category of small-molecular modification. Surfactant adsorption is an effective method for CNCs’ modification, which can meet the needs of some specific scenarios.

#### 3.1.5. Amidation and Imidization

**Figure 16:**
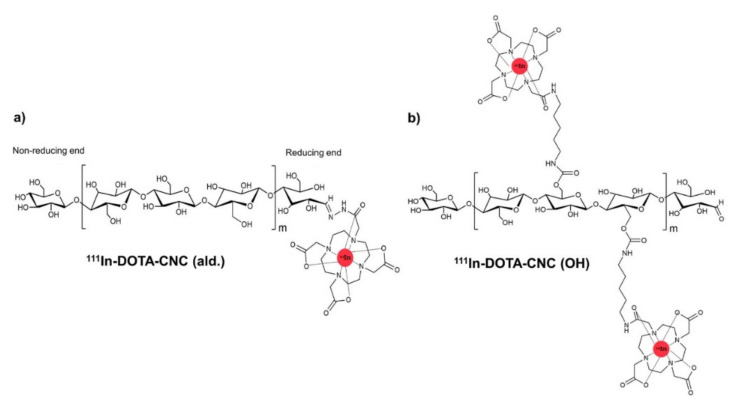
Schematic representations of (**a**) ^111^In-DOTA-CNC (ald.) and (**b**) ^111^In-DOTA-CNC (OH) [75]. Publisher: Royal Society of Chemistry.

Imlimthan et al. realized the covalent assembly of macrocyclic chelating agent DOTA (1,4,7,10-tetreaazacyclododecane-1,4,7,10-tetraacetic acid) on CNCs [74]. Firstly, DOTA was bonded to form DOTA-NH_2_, conjugated to the hydroxyl groups on the CNC surface through N, N’-carbonyldiimidazole (CDI)-mediated amide coupling reaction. CDI was carried on using as an activator. The aldehyde groups (C-H=O) at the reducing end of CNC were proven capable of reacting with CDI (CNC-CDI) and DOTA (CDI-DOTA), respectively (Figure 16). The reaction happens under mild conditions, such as in an anhydrous dimethyl sulfoxide solvent, at 50–60 °C, under an argon atmosphere, which is harmless to CNCs’ original morphologies. Moreover, the terminal aldehyde modification appears appealing as it makes the OH-groups on the CNCs’ surface accessible for other subsequent functionalization and payload incorporation.

**Figure 17:**
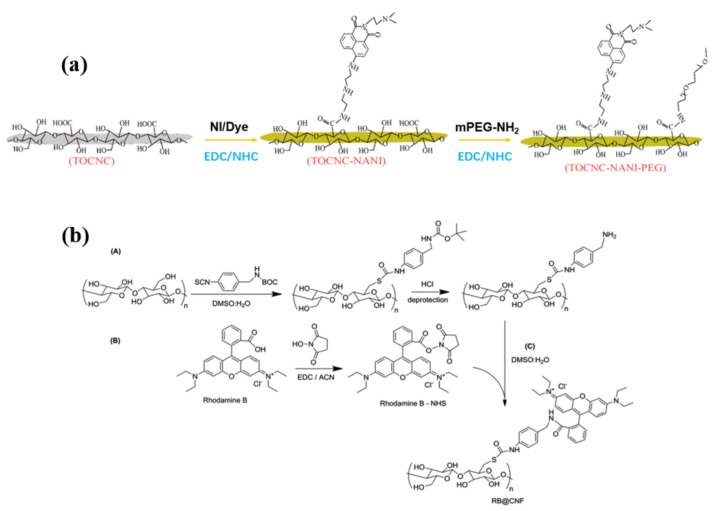
(**a**) Synthesis route of fluorescent PEG-modified TOCNC [55]. (**b**) Fluorescent labeling of cellulose nanofibrils (**A**)(CNF) with rhodamine (**B**) [75]. (**a**) © 2020 Elsevier B.V. All rights reserved. (**B**) open access.

**Figure 18:**
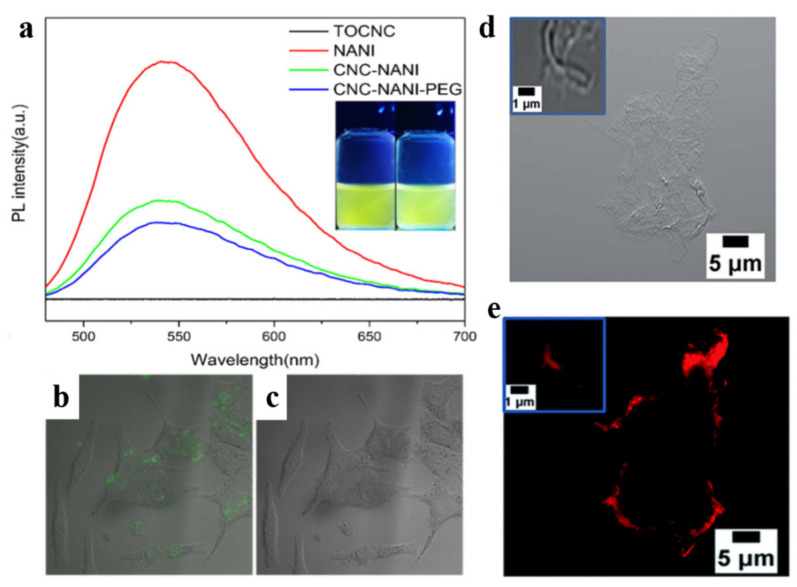
(**a**) Spectra of NANI, TOCNC, TOCNC-NANI, and TOCNC-NANI-PEG in an aqueous solution. The fluorescence photographs (inset) of TOCNC-NANI (**left**) and TOCNC-NANI-PEG (**right**) [55]. Confocal microscopy images of Hela cells labeled with TOCNC-NANI-PEG (5.5 × 10^−3^ g/L), (**b**) overlay, (**c**) bright field [55]. Fluorescence (**e**), brightfield (**d**) of rhodamine B@CNF hybrids. The inset of (**d**) shows a high magnification image of a strand of labeled CNF [75]. © 2020 Elsevier B.V. All rights reserved.

Synthesis of fluorescent CNCs with good water dispersibility was reported by coupling a fluorescent 1,8-naphthalimide dye and biocompatible poly(ethylene glycol) (PEG) to CNCs [55]. The fluorescent dispersive tails were grafted onto CNCs through a two-step ethylene dichloride (EDC)/N-hydroxysuccinimide (NHS)-mediated coupling reaction (Figure 17a). For this reaction, the dye-labeled modified CNCs were synthesized by carbony (-COOH) abundant on CNCs in an aqueous environment. The requirement for this soft environment is that EDC/NHS reaction happens at the pH of 5.2 with PBS buffer solution. Stirring for 18 h in the dark at room temperature also helps to maintain the original morphologies of CNCs. The dye-CNC modified by PEG brush has strong yellow-green fluorescence emission. The authors proved that the fluorescent CNC probe could penetrate the cell membrane, be evenly dispersed in the cell, and have good biological imaging performance (Figure 18b).

Similarly, Navarro et al. used the amidation reaction between amine-modified cellulose and N-hydroxysuccinimide (NHS)-modified rhodamine B to graft fluorescent dye rhodamine B onto cellulose [75]. Li et al. reported the synthesis of novel, multi-stimuli responsive, and reversibly fluorescent cellulose derivatives containing thiol groups and rhodamine spiroamide, and the already formed nanoparticles can be dissolved again under redox-controllable conditions [76]. CNCs have a high specific surface area, good hydrophilicity, and biocompatibility, and their high aspect ratio and acute angle facilitate entry into cells and organisms [77,78]. These biocompatible nanoparticles will play an important role in biomedicine, such as cancer diagnosis, drug delivery, and biological imaging, etc [79,80,81]. 

### 3.2. Macromolecules Chemically “Grafted from” CNCs

Macromolecules are molecules with large relative molecular mass, and complex structure: carbohydrates, nucleic acids, proteins, and lipids belong to this. Except for a change in interfacial properties of CNCs by the small molecules, it brings additional properties such as optics, biology, and electromagnetics by modifying the surface of CNCs with macromolecules.

**Figure 19:**
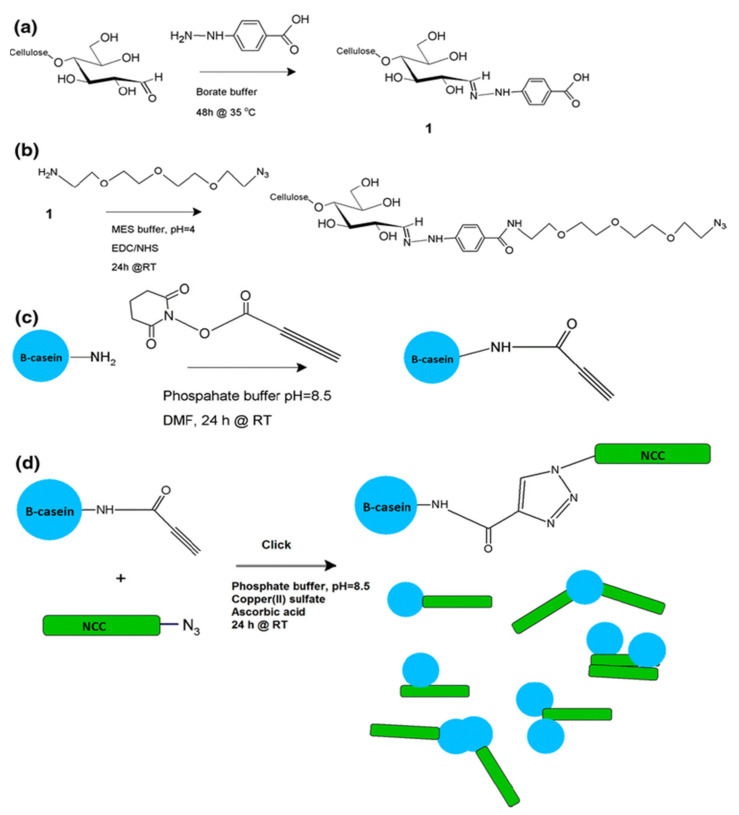
Schematic representation of sequential reactions, (**a**) activation of CNCs’ reducing end groups with carboxyl groups, (**b**) azide functionalization of CNCs’ reducing ends by carbodiimide-mediated coupling reaction, (**c**) acetylene functionalization of β-casein, and (**d**) the click reaction between azide functionalized CNC and acetylene functionalized protein [85]. Copyright © 2013, Springer Science Business Media Dordrecht.

**Figure 20:**
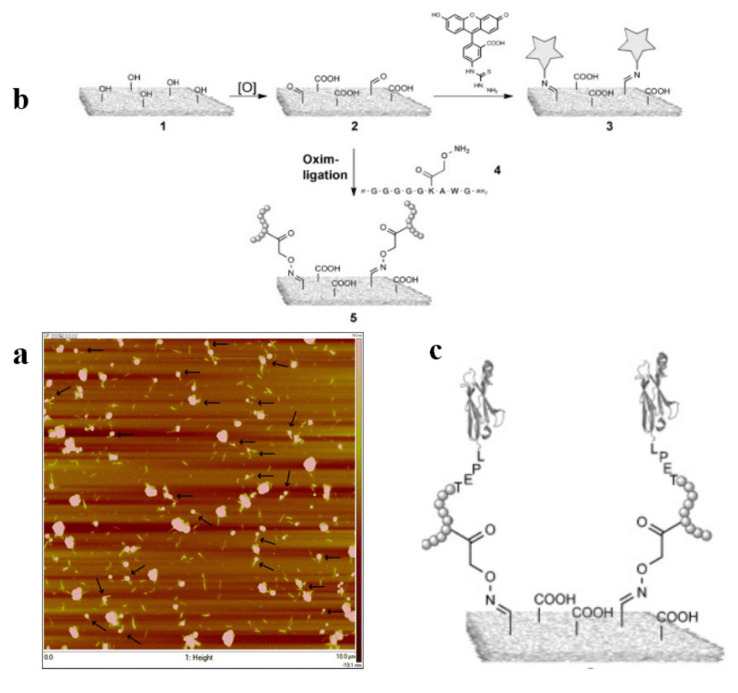
(**a**) AFM height image (10 × 10 μm) of CNC-protein hybrid nanoparticles (arrows used to highlight mushroom-like nanoparticles) [85]. (**b**,**c**) General synthesis strategy. The CNC surfaces with their functional groups are shown as gray areas, fluorescein groups as stars (**b**) [86], peptide 4 in the one-letter code and shown schematically as a chain (**c**) [86]. (**a**) Copyright © 2013, Springer Science Business Media Dordrecht. (**b**,**c**) WILEY‐VCH Verlag GmbH & Co. KGaA, Weinheim

Introducing biomolecules on CNCs’ reducing ends is a challenge. However, it can lead to directed self-assembly of CNC into high-order nanomaterials if achieved in a controlled manner [77,82,83,84]. Karaaslan et al. performed a copper-catalyzed 1,3-dipoles “click” reaction between CNCs with azide (–N_3_) tail and protein micelles with amine groups (-NH_2_) [85]. At the reducing end of CNC, the carboxylic acid group reacts with the amine group of the bifunctional azide compound through a carbodiimide-mediated coupling reaction. This study proves a possibility of attachment of macromolecular-sized protein, inspired by other biological membranes, DNA and RNA. The carbon chain of CNC also endorsed the functionalization of CNCs. Macromolecular arrangements can be expected [85] (Figure 19). Likewise, Christina Uth et al. also successfully converted the primary alcohol on the CNC surface to aldehyde, and a bioactive module can be fixed to the CNCs by coupling with oxime (Figure 20) [86]. Thence, the biological properties of the coupling compound can be combined with the advantages of cellulose, such as biocompatibility, low cytotoxicity, and nanometer size.

### 3.3. Polymers Chemically “Grafted” on CNCs

**Figure 21:**
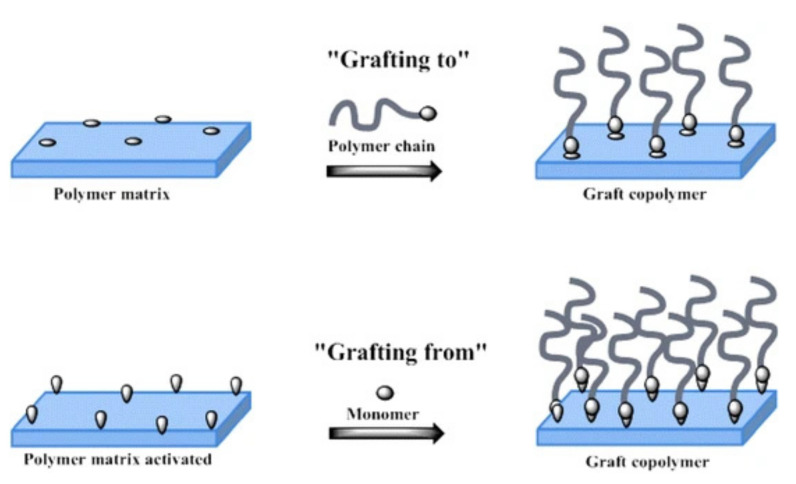
Synthesis of polymer brushes using “grafting to” and “grafting from” approaches [92]. Copyright © 2016, Springer International Publishing Switzerland.

Extra functionalities can be obtained by modifying CNCs with polymer, e.g., good film formability, high stability, well responsive wettability, optical information storage, and optical switching with the binding of polymers. In the method of polymer modification, “grafting” is a promising technology that can be used to introduce special functional groups to modify CNC’s original properties and expand its scope of application [87,88,89] The process of “grafting to” and “grafting from” are two different ways to change the surface chemical and physical properties of CNCs and its derivatives (Figure 21). “Grafting to” refers to first synthesizing a side chain with a reactive functional group and then introducing a polymer side chain to the CNCs through a chemical reaction between the two functional groups. This method can well control the molecular weight distribution of the main and side chains of the polymer; however, as the grafting reaction proceeds, the steric hindrance between the branches increases as the graft density increases [90,91].

“Grafting from” refers to first synthesizing CNCs with initiating active center and then initiating the polymerization of the second monomer through the active center on CNCs [76,93]. This method allows us to control the main chain’s molecular weight distribution and obtain a higher graft density [94]. During the reaction, “Grafting from” has a significant selectivity for monomers, and the structure is easy to adjust and control. However, there may be a free-radical coupling phenomenon within or between the molecules that affects the polymer molecular brush size and structural uniformity [95]. A commonly used polymerization method is atom transfer radical polymerization (ATRP), as discussed below [96,97].

#### 3.3.1. “Grafting from”

**Figure 22:**
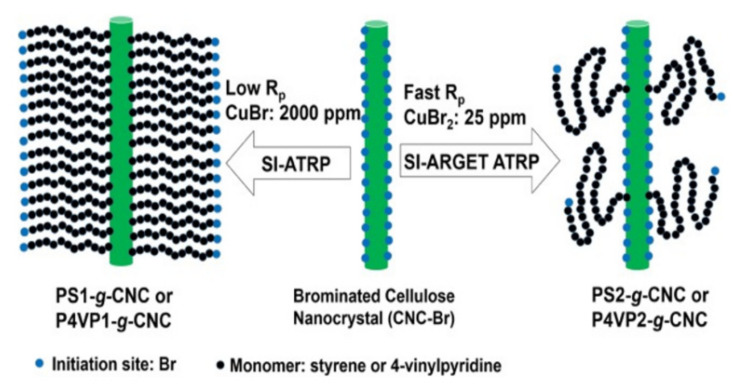
Schematic representation of the proposed mechanism to account for the differences found with SI-ATRP and SI-ARGET ATRP from CNC [100]. 2018 Elsevier Ltd. All rights reserved.

**Figure 23:**
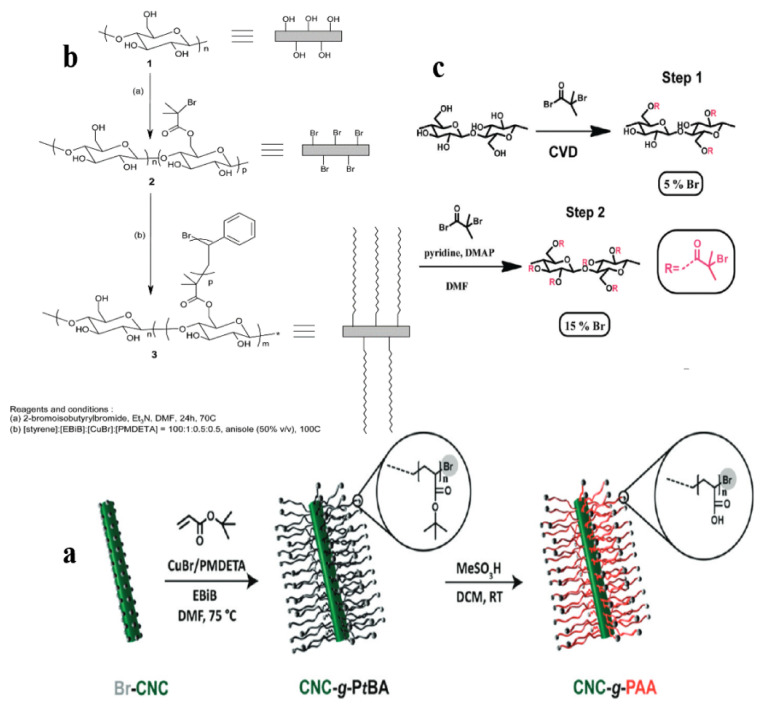
(**a**) Reaction scheme for Cu-mediated SI-CRP of PtBA, followed by acid hydrolysis of the tertiary alkyl functionality to yield PAA brushes grafted from the CNC surface. (**b**) Synthesis of polysaccharide nanocrystals-g-polystyrene by ATRP [99]. (**c**) Initiator modification of CNCs. Two-step reaction scheme for CNC surface esterification: Step 1, in the gas phase (CVD) for partial and Step 2, in DMF for high initiator surface grafting. Copyright © 2009, American Chemical Society.

The “Grafting from” approach gives CNCs a higher graft density, but only if the CNCs have a high active site. There has been a large number of reports using 2-bromoisobutyl bromide (Br-iBBr) as an ATRP active initiator on the CNCs surface [97,98], which ensures a high esterification rate. As early as 2009, G. Morandi et al. esterified CNCs through the Br-iBBr activated atom-transfer radical-polymerization (SI-ATRP) reaction (Figure 23b) [99]. Zhang et al. conducted surface-initiated ATRP and electron-transferred ATRP to regenerate surface-initiated activator (SI-ARGET ATRP) to graft polystylene polymers on CNCs (PS-g-CNC) surfaces [100]. Grafted polymer chain of the SI-ARGET ATRP method is longer, but the graft density is lower than that of SI-ATRP polymer (Figure 22). The authors proposed that both CuBr and CuBr_2_ were good in initiating the ATRP reaction. The dose and type of “Grafting from” determine the chain density with a certain surface area. The SI-ARGET ATRP is more conducive to grafting mushroom-like polymers on CNCs, and traditional SI-ATRP is more suitable for synthesizing polymer brushes on CNCs.

Those mentioned reactions above adopted a wet solvent treatment to activate CNCs with Br-iBBr as the grafting site. Interestingly, chemical vapor deposition (CVD) proves better to avoid the re-dispersion of dried CNCs in organic solvents (Figure 23a). Firstly, the CNCs were kept in a gas phase (CVD), and it was partially brominated with Br-iBBr at 5% Br content. Subsequently, they put the CNC-Br in DMF for high initiator surface grafting. This step fully functionalizes the hydroxyl functional groups on the CNCs’ surface giving a 15% Br content. They confirmed that the initiator groups’ density on the CNCs’ surface was 4.6 bromine ester groups/nm^2^, effectively ensuring a very high ATRP initiator on the CNCs surface. Hence, this is a solid foundation for synthesizing dense polymer brushes, such as poly(tert-butyl acrylate) (PtBA) and poly(acrylic acid) (PAA). Except for the activator Br-iBBr, Majoinen et al. also used a copper (Cu)-mediated surface-induced controlled radical polymerization (SI-CRP) to synthesize PAA-linked CNCs of different lengths [101] (Figure 23a). First, a well-defined high graft density PtBA brush was synthesized on the Br-CNC surface obtained by pretreatment, and then their tertiary alkyl functional groups were hydrolyzed with acid, which helps to switch the wettability of CNCs effectively. 

**Figure 24:**
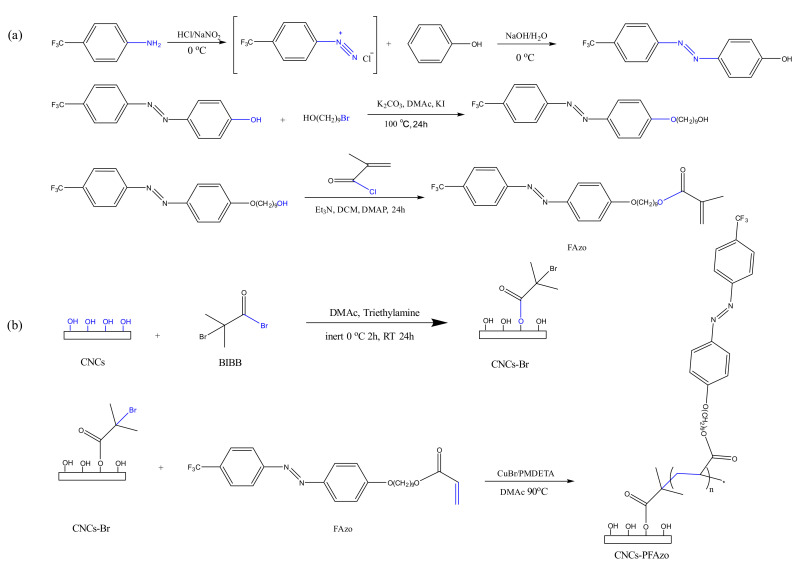
Synthesis of (**a**) FAzo-monomers and (**b**) poly-FAzo brushes on CNC [102]. open access.

**Figure 25:**
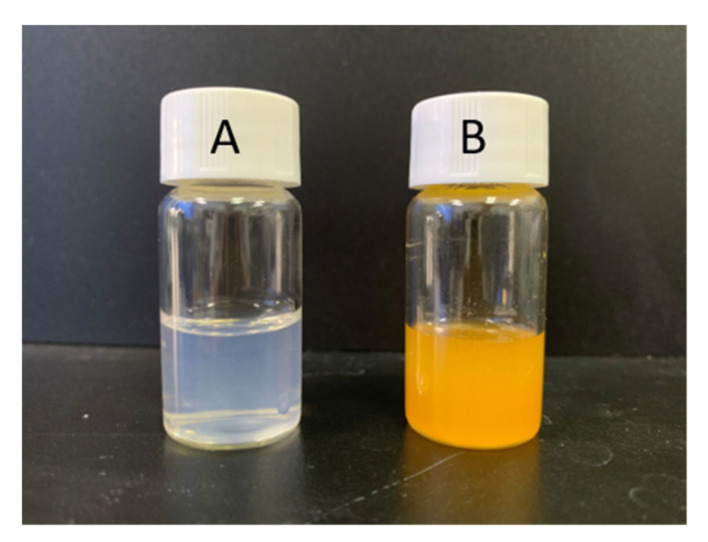
Photograph of two separate CNC samples: (**A**) Aqueous suspension of CNC and (**B**) dimethyl sulfoxide suspension of the CNC-FAZO [102]. open access.

A highly hydrophobic heterocyclic azo-benzodiazepines CNC derivative was synthesized via graft copolymerization by Xu et al. [102]. They first functionalized CNCs with α-bromoisobutyryl bromide. The stepwise graft copolymerization modification of CNCs was displayed in Figure 24. 9-[4-[2-[4-(trifluorometh) phenyl] diazenyl] phenoxy] nonayl acrylate (FAzo) was used to prepare CNC-FAzo with around eight monomers. Homogeneous and yellow-colored FAzo-grafted CNCs suspension was well dispersed in a dimethyl sulfoxide solvent (Figure 25). The reaction is facile and will have massive potential in optical information storage, optical switching, and linear optics.

**Figure 26:**
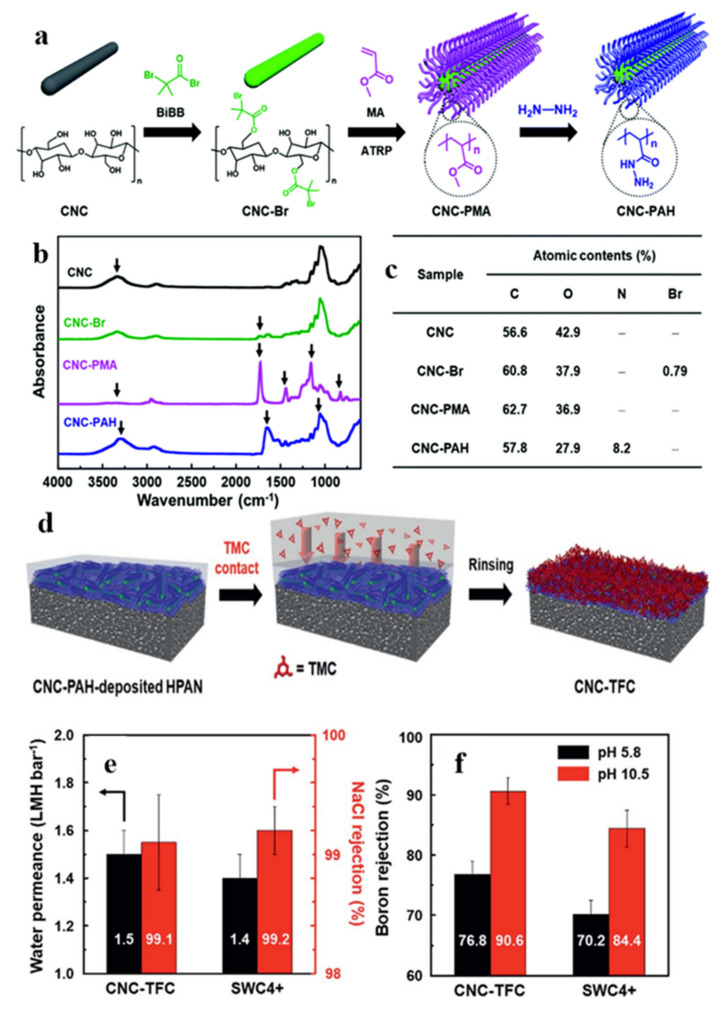
(**a**) Schematic of the synthesis of the PAH grafted-CNC (CNC-PAH) particles. (**b**) FT-IR spectra and (**c**) XPS chemical composition of pristine CNC, CNC−Br, CNC-PMA, and CNC-PAH. (**d**) Schematic of the TFC membrane’s fabrication (CNC−TFC) using the CNC−PAH particles via the LIP technique. (**e**) Water permeance and NaCl rejection, (**f**) boron rejection at pH values of 5.8 and 10.5 of the CNC-TFC and commercial RO (SWC4+) membranes [103]. Publisher Royal Society of Chemistry.

Park et al. adopted a method of layered interfacial polymerization (LIP) to design a core-shell structured poly (acryloyl hydrazide) (PAH) grafted cellulose nanocrystal (CNC-PAH) as a new building material for use in reverse osmosis membranes (CNC-TFC) (Figure 26) [103]. Prepared CNC-TFC has high selectivity and a low fouling rate. Many amine groups in high-density CNC-PAH provide high-density reaction sites for trimesoyl chloride (TMC), resulting in a highly cross-linked ultra-thin selective layer structure. Functionalized CNC derivatives have high boron trapping ability, which hinders boron from passing through the (CNC-TFC) membrane, enhances the boron removal ability, and solves the commercial TFC’s drawback that boron rejection rate is not high. Boron is a harmful solute and will make the TFC membrane easy to scale and shorten the service life of the TFC membrane. Therefore, the increase in the boron rejection rate improves the surface finish of the CNC-TFC membrane. The hydrophilic and highly functional nature of the CNC-TFC membrane makes it have higher water permeability, making the CNC-TFC membrane superior to industrial membrane’s organic dirt resistance.

**Figure 27:**
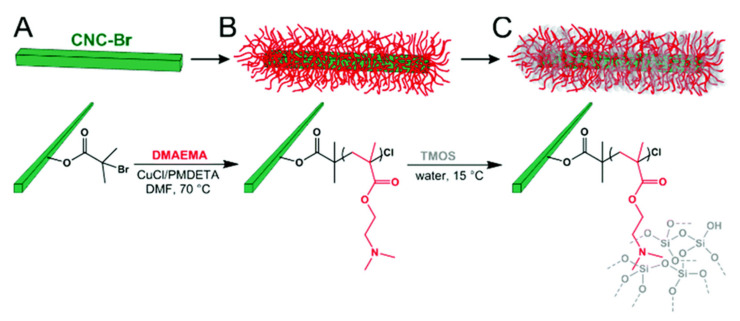
(**A**) Surface-modified CNC-Br is grafted with PDMAEMA brushes via SI-ATRP to yield (**B**) CNC-g-PDMAEMA core-shell templates that are used as (**C**) nanoreactors for the selective mineralization (hybrid formation) of silica within the PDMAEMA brush shell (SiO2@CNC-g-PDMAEMA) [104]. Royal Society of Chemistry.

Morits et al. explored the potential applicability of the prepared polymer brushes and developed an effective template-oriented synthesis [104]. The method synthesizes porous inorganic nanorod templates with adjustable diameter and aspect ratio using CNC-based polymer brushes (Figure 27). This method is to graft PDMAEMA brush with CNCs, and then form SiO_2_ network in the polymer brush layer to achieve the practical template orientation synthesis of microporous and mesoporous silica nanorods. The subsequent calcination produces highly porous and hollow nanorods, which can be used for insulating aerogels, catalyst supports, or storage and delivery systems.

The ATRP is a typical “grafting from” method, except that Andrés Alanis et al. also developed a new reaction condition. Based on plasma-surface modification, reaction on CNCs generates free radicals through molecular ionization and glows discharge and then initiates a polymerization reaction on the surface in contact with the plasma [105]. Polycaprolactone, polystyrene, and polyfarnesene were successfully grafted on the CNCs by this method, and the resulting product did not change the rod-like structure of CNCs. It is found that the mechanical properties of CNCs composite can be high after adding modified ABS (acrylonitrile-butadiene-styrene) additive, and the impact resistance was 114% higher than that of ABS polymers without additive, which indicates that the compatibility of CNCs has been improved by plasma-induced polymerization surface modification.

For the surface modification of CNCs, the reaction conditions must be fierce enough to react with the surface hydroxyl groups while not reacting with the internal -OH of the cellulose and not affecting its intrinsic properties, especially size crystallinity and original mechanical properties. However, so far, the ATRP reaction still has its limitations, such as the fact that, the CNCs have gone through multiple times of drying step in order to fit in the anhydrous dosing of BiBB initiator, which brings in severe aggregation. The aggregation caused by the drying process is not conducive to subsequent treatment and is not desirable. Many essential attributes such as chirality associated with the individuation have already disappeared. Despite that, the “grafted from” products of PS, PAA, PDMAEMA, and poly-azobenzene derivative polymers can have huge potential in the field of biomedicine, from drug delivery, antioxidants, antimicrobial, and so on [106].

**Figure 28:**
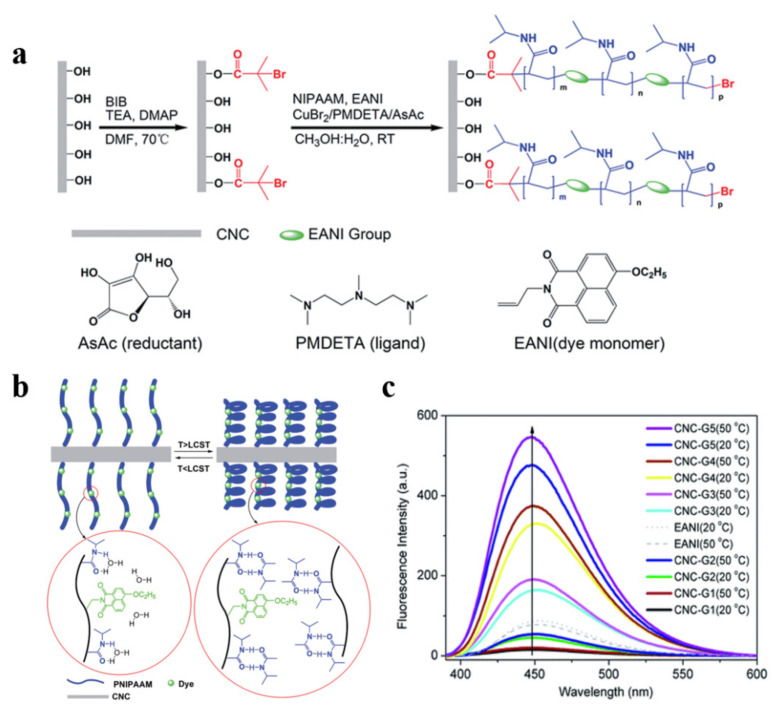
(**a**) Synthesis route for the immobilization of the initiator on CNCs and subsequent surface grafting of poly (NIPAAM-co-EANI). (**b**) Conformation of grafted PNIPAAM brushes below the LCST and above the LCST. (**c**) Fluorescence emission spectroscopy of surface-grafted CNCs (0.02 wt% in H_2_O) and EANI (10^−6^ M in H_2_O) [107]. Royal Society of Chemistry.

The thermo-responsive polymer was successfully demonstrated capable of being “grafting from” CNCs by Wu et al. [107] The prepared CNCs were grafted with a thermally responsive poly(N-isopropylacrylamide-) (PNIPAAM). The fluorescent part, 4-ethoxy-9-allyl-1, 8-naphthalimide (EANI), was also co-polymerized with PNIPAAM. The success of the polymerization was still guaranteed by using the two-component solvent system of CH_3_OH/H_2_O in the mixture with different volume ratios (Figure 28a). Due to the thermally driven chain dehydration of the grafted PNIPAAM brush, the obtained surface-grafted CNCs showed enhanced fluorescence (Figure 28c). 

When EANI was attached to CNCs with PNIPAM, the collapse of the copolymer brushes at higher temperatures inhibited the rotation and vibration of dye molecules, decreased the dye’s nonradiative transition, and increased its fluorescence intensity. This change in physical interaction is shown in Figure 28b. This thermo-responsive system could function as a drug carrier, a fluorescent probe, or a sensor [108,109].

#### 3.3.2. “Grafting to”

**Figure 29:**
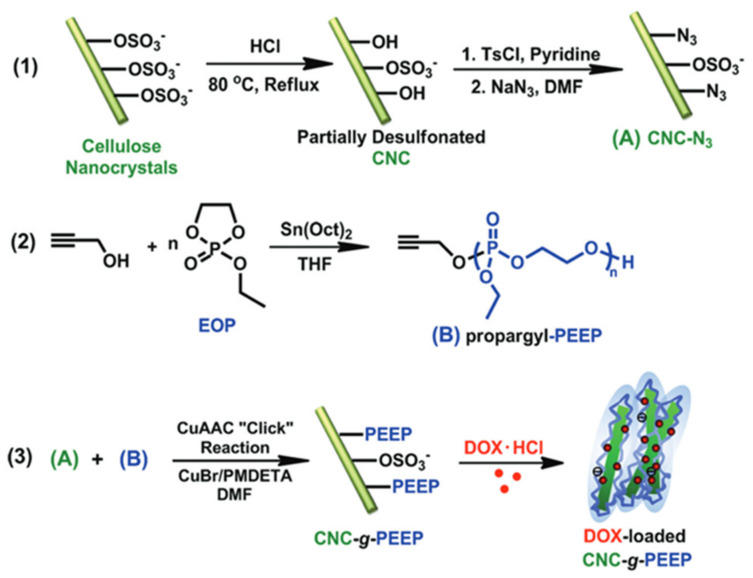
Schematic illustration of the synthesis pathway of CNCs−g−PEEP via (CuAAC) “click” reaction and the formation of positively charged doxorubicin-loaded CNC [110]. Royal Society of Chemistry.

Wang et al. used a “grafting to” method to modify CNCs with biodegradable polymers [110]. Propargyl-terminated poly (ethyl ethylene phosphate) (propargyl-PEEP) was synthesized by ring-opening polymerization and subsequently grafted onto azide-modified CNC nanoparticles through Cu-catalyzed azide-alkyne cycloaddition (CuAAC) “click” chemistry (Figure 29). The highly negatively charged modified nanoparticles could be used to load doxorubicin via electrostatic interactions.

**Figure 30:**
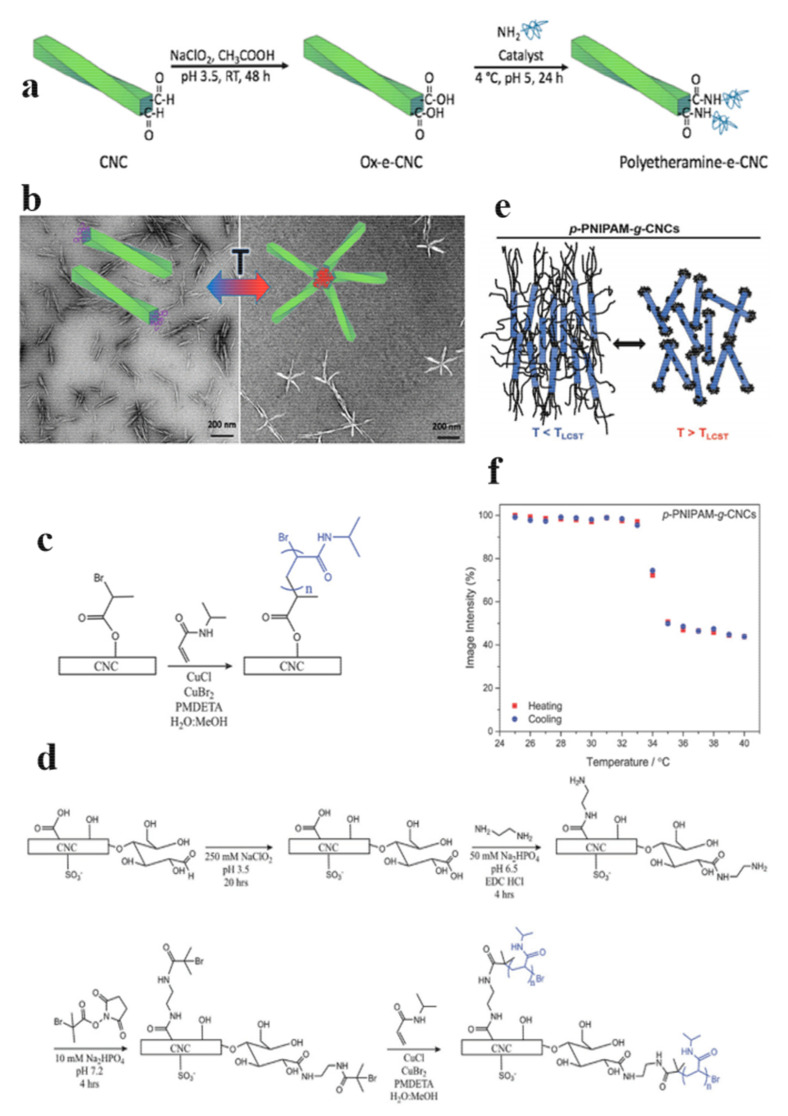
(**a**) Two-step asymmetric functionalization of CNCs with thermosensitive polyetheramine chains in aqueous mediums and (**b**) thermoreversible association of polyetheramine-e-CNCs into star-shaped assemblies [111]. (**c**) “Brush” SI-ATRP of PNIPAM from CNCs [114]. (**d**) “Patchy” SI-ATRP of PNIPAM from CNCs [114]. (**e**) Schematic illustrating of the proposed temperature-responsive assembly in aqueous dispersions of p -PNIPAM-g-CNCs and b -PNIPAM-g-CNCs below and above the LCST of PNIPAM [114]. (**f**) Image intensity of polarized optical microscopy images of 10 wt% p-PNIPAM-g-CNCs as a function of temperature during a 1 °C min^−1^ heating and cooling cycle [114].(**a**,**b**) Copyright © 2019, American Chemical Society. [114] © 2018 WILEY-VCH Verlag GmbH & Co. KGaA, Weinheim.

To date, only a few works have focused on the asymmetric modification of the CNCs tips. Lin et al. developed a water-tolerant synthetic protocol to selectively functionalize only the terminal end to prepare CNCs with end-tethered polymer chains [111] (Figure 30a,b). The hemiacetal end group was first oxidized to carboxylic acid, and then the carboxylic acid was modified with an ATRP initiator, followed by polymerization [112]. Although this pathway was prone to side reactions, this approach opens up new opportunities to create hierarchical structures [113]. The authors confirmed that the dispersed modified CNCs assembled, forming the arms of regular four-, five-, or six-branched stars.

Zoppe’s group also synthesized poly (N, N synthesized methacrylic acid-dimethylaminoethyl methacrylate) (PDMAEMA) grafted CNCs, and studied liquid crystal formation in aqueous suspension (Figure 30e) [114]. The authors synthesized “Patchy” PNIPAM-modified CNCs and “brush” PNIPAM-modified CNCs according to schemes 1 and 2 (Figure 30c,d). They found the thermal switching observed for the “patchy” PNIPAM-modified CNCs that was unprecedented and possibly useful for sensing and smart packaging applications.

**Table 2:** Representative functional modification of cellulose nanocrystals.

Types of Modification	Method	Findings	Advantages	Limitations	Ref.
Small molecule functionalized CNCs	Grafted with lactic acid (CNC-g-LA)	High surface graft density, reduce the graft length to preserve the original size and morphology of CNCs	Green, simple, one-step; good dispersion in chloroform	Bad dispersion in water, ethanol, acetone, THF, toluene	Wu et al. [62]
	Zinc phthalocyanine conjugate functionalization	Exhibited absorption and emission maxima at 690 and 715 nm	Photoluminescence-based technologies	Cost, activation of linker	Alam et al. [66]
	Aldehyde functionalization	DOTA-NH_2_ linked to the aldehyde	Makes the OH-groups on the CNC surface accessible for other subsequent functionalization	Low reactivity	Imlimthan et al. [74]
Macromolecule Functionalized CNCs	Polydopamine functionalization	Contact angle shift from 21 to 95.6°	Spontaneous clicking reaction	Low hydrophobicity	Shang et al. [65]
	CNCs grafted biomacromolecule	Click reaction between azide functionalized CNC and acetylene functionalized protein	Biocompatibility, low cytotoxicity, and nanometer size	Strict reagent selection due to protein’s nature	Karaaslan et al. [85]
	ATRP polymerization functionalization followed by mineralization of silica	Graft PDMAEMA brush with CNCs, form SiO_2_ network in the polymer brush layer to achieve template	Forms microporous and mesoporous silica nanorods	Severe aggregates during the solvent exchange for CNC	Morits et al. [104]
	Azo-benzodiazepines functionalization	Hydrophobic	Massive potential in optical information storage, optical switching, and linear optics.	Difficulties in purification and redispersion	Xu et al. [102]
	Aldehyde functionalization with polyetherimide	Forms arms of regular four-, five-, or six-branched stars	Creates hierarchical structures	Few reactivity sites	Lin et al. [111]

### 3.4. Drawbacks and Future

Small molecular and macromolecular level surface modifications on the CNCs play a vital role in the surface/interfacial and functional properties of CNCs. Small molecular lactic acid, UV-light absorbing PABA, alcohols, dye, protein, PEG, polydopamine, and other functional polymers were proven to be successfully attached to the CNCs. Except for changes in the chemical composition, these active substances bring the CNCs more functionalities, e.g., hydrophobicity, hierarchical structures, optical light tenability, biological carrier, selective light absorption, and so on. However, it is also easy to note some drawbacks as well.

Based on the different origins of CNCs, researchers did not have a product standard on surface charges, fiber aspect ratio, size distribution, and so on. Under the circumstances, it is tough to propose/repeat/continue a standard surface modification protocol. Often, researchers fail to repeat the same procedure straightforwardly. For future development, it is urgent to publish a globally accepted standard for the CNCs product itself.

Concerning types of necessary surface modifications, it is essential and necessary to tackle the solvent exchange problem as well. Chemical modification of CNCs is usually required to be performed in a non-aqueous medium, and as CNCs are present in an aqueous medium, the aqueous medium should be removed and replaced with a non-aqueous medium without aggregation of nanocrystallites. Currently, there are three standard methods to obtain a CNC suspension in a non-aqueous solvent. The first method is solvent replacement by mixing the required non-aqueous solvent and CNC aqueous suspension and evaporating the aqueous medium. The limitations of this method are the mismatched boiling point of the solvent, recycling difficulties, and difficulty in removing bonded water in the CNCs. Except this, a free-drying approach is also often used to remove the aqueous medium in CNC suspensions. However, if we freeze-dried and obtained the CNCs powder, it is challenging to re-disperse it in the target organic solvent again. A long operating time and high energy consumption are the drawbacks of this technique. Other drying techniques, such as spray drying, can produce individualized particulates, but the spray drying technique has operation cost and maintenance issues.

The drawbacks of each surface modification depends on the desired application between the small molecule and macromolecular level modification type. We may choose which one suits our aim. For instance, when we consider improving hydrophobicity, polymerization on CNCs is often better than the small molecule bonded method. This is because the macromolecule type of polymerization coats a much thicker plastic layer on the CNC, which guarantees the high stability of the mixture. For the surface modification of CNCs, it is still unclear whether the surface modification is in the C6 position or others for each modification. The effects on specific reaction mechanisms and the interactions with the liquid medium are not investigated yet.

Nevertheless, while we look into the future in the nanocellulose field, it is generally admitted that the nature biopolymer CNC has a vast potential, e.g., to replace the petroleum oil-derived polymers partially. We still need to dissolve the technical problems and put more effort into these areas in the future. For example, we need to publish a globally recognized CNCs product standard, a good liquid environment (e.g., solvent) before and after CNC’s surface modification, reaction mechanism, as well as the practical applications in high value ends. Researchers are looking forward to reducing the use of decomposable plastics, trying to protect the sea animals, plants, and ourselves. Therefore, the innovation of CNC for functional derivatives seems undoubtedly meaningful.

## 4. Conclusions and Outlooks

As an essential step, CNCs derivatives will be applied in various applications in the future, such as filler, drug delivery, optical scattering film, solar panel support substrate, intelligent coating, and LED display devices. The research on CNCs derivatives has excellent potential, and the methods to build up more functional and diverse derivatives with interspersing designation are worth further exploration in the future. The key is the tailored surface modification of cellulose nanomaterials. In the last few years, a multitude of surface functionalization techniques have been explored to alter the surface structure without losing structural integrity. Traditionally extracted from multiple cellulose sources via sulfuric acid hydrolysis, these cellulosic nanomaterials are modified for a wide range of applications, briefly introduced in this review. This review especially focuses on summarizing the surface chemical modification methods of CNCs with different molecule’s levels, i.e., small molecule derivatization modification and grafting of functional macromolecules.

Advancing the chemical modification of cellulose nanocrystals is challenging. Despite extensive efforts in CNC surface modification, many challenges prohibit their large-scale implementation and commercial use. As far as CNC production is concerned, the mineral acid hydrolysis method to produce CNCs is the most popular way, especially sulfuric acid hydrolysis. However, there are still some disadvantages in this method. First, the corrosion problem of the equipment is serious, which leads to the insufferable cost of production. Furthermore, a large amount of waste acid and other pollutants will be produced in the process of acid hydrolysis, which are not easy to dispose of and recover. Thus, it is still challenging to achieve industrial production at a high capacity, which will be one of the bottlenecks restricting the broad application of CNC due to environmental pollution, yield, cost, and other factors. These methods generally suffer from one or more of the following challenges:

(1) How to modify CNCs in a more environmentally friendly way while maintaining the surface properties of the crystal, since most modifications use expensive and non-sustainable reagents.
(2) How to modify CNCs more efficiently while maintaining the surface properties of the crystal, since most modifications need multiple steps (including solvent exchange and initiator attachment) and due to the low yields of modified CNCs.
(3) How to retain its original size and crystal structure after multiple times of drying and cleansing.
(4) How to keep uniform products after using large volumes of organic solvents in which CNCs are not colloidally stable.

Overall, in the next 10 to 20 years, we can expect more characteristics of this unique and attractive material, but also foresee that CNC derivatives with functions can be applied in many areas. Based on the scenario that we may solve related to scientific and technological problems in the field, we may target and retain the desired attributes for the next generation of applications.

## Figures and Tables

**Table 1:** Hydrolysis conditions and characteristics of CNC from different sources.

Sample	Bamboo [16]	Eucalyptus [16]	Sisal [16]	Curauá [16]	Lemon Seeds [46]	Tomato Peel [47]	Doum Tree [48]	Sugar Palm [49]	Cotton Fiber [50]
**Hydrolysis conditions**									
Acid-fiber ratio (mL/g)	10/1	9/1	15/1	15/1	20/1	8.75/1		20/1	11/1
Temperature (°C)	60	50	50	50	45	45	50	45	50
Hydrolysis time (min)	12	50–65	30	45	90	30	30	45	45
H_2_SO_4_ concentration (wt%)	65	65	65	65	64	64	64	60	63.9
Sonication time (min)	4	1	7	7	30	5	5	30	-
**Characteristics of CNCs**									
Yield (%)	30	17	9	-	27.61	15.7		29	41.7
Sulfur content (% S) ^a^	1.04	0.96	0.53	0.95	-	0.48			
Total anionic sites (mmol kg^−1^) ^a^	324	275	166	297	-	-	-	-	-
Crystallinity index (%)	-	-	-	-	69.67	80.8	90	85.9	91.26
Zeta potential (mV)	−59 ± 2	−48 ± 7	−49 ± 2	−52 ± 1	−40.27	−52.4 ± 1.4		−61.50 ± 1.65	
Length (nm) ^b^	100 ± 28	100 ± 33	119 ± 45	129 ± 32	145 ± 20.7	135 ± 50	450	130 ± 30.23	140.9
Width (nm) ^b^	8 ± 3	7 ± 1	6 ± 1	5 ± 1	18.5 ± 6.5	7.2 ± 1.8	5.3	9 ± 1.96	-
Height (nm) ^c^	4.5 ± 0.9	4.5 ± 1.0	3.3 ± 1.0	4.7 ± 1.0	-	3.3 ± 1.2		-	-
Aspect ratio	22	22	36	27	8	21	85	15	-

^a^ Sulfur content and total anionic sites result from conductometric measurements, ^b^ Length and width values result from TEM observation, ^c^ Height result from AFM experiments.
